# ReLink strategy in diagnosed-but-untreated hepatitis C-positive patients in Germany: report from a single center: Hepatitis C ReLink project Germany

**DOI:** 10.1055/a-2349-2767

**Published:** 2024-07-16

**Authors:** Sarah Lange, Christina Baehr, Nur Irem Cakman-Hinrichs, Katharina Cron, Hannah Fengels, Christina Gregor, Katrin Matschenz, Jörg Petersen, Robin Steinfurth, Albrecht Stoehr, Stefan Unger, Maria Gil Mir, Candido Hernández, Marianna Schwenken, Peter Buggisch

**Affiliations:** 1 MVZ ifi-Institut GmbH an der Asklepios Klinik St. Georg, Haus L, Hamburg, Germany; 2 MVZ ifi-Institut GmbH An der Asklepios Klinik St. Georg, Haus L, Hamburg, Germany; 3 MVZ ifi-Institut GmbH An der Asklepios Klinik St. Georg, Haus L, Hamburg, Germany; 4 MVZ ifi-Institut GmbH An der Asklepios Klinik St. Georg, Haus L, Hamburg, Germany; 5 MVZ ifi-Institut GmbH An der Asklepios Klinik St. Georg, Haus L, Hamburg, Germany; 6 MVZ ifi-Institut GmbH An der Asklepios Klinik St. Georg, Haus L, Hamubrg, Germany; 7 MVZ ifi-Institut GmbH An der Asklepios Klinik St. Georg, Haus L, Hamburg, Germany; 8 MVZ ifi-Institut GmbH An der Asklepios Klinik St. Georg, Haus L, Hamburg, Germany; 9 Former employee of Gilead, München, Germany; 10 65847 Gilead Sciences Europe Ltd, Uxbridge, United Kingdom of Great Britain and Northern Ireland; 11 60043 Gilead Sciences GmbH, Martinsried, Germany

**Keywords:** Hepatitis C elimination, ReLink strategy, diagnosed-but-untreated patients, linkage to medical care, patients lost to follow-up, difficult-to-treat person, Beseitigung von Hepatitis C, ReLink-Strategie, Diagnostizierte aber unbehandelte Hepatitis C-positive Patient*innen, Rückführung in die medizinische Versorgung, Der Nachbeobachtung verloren gegangene Patient*innen, Schwierig zu behandelnde Personen

## Abstract

**Objective** The ReLink project aims to reintegrate diagnosed-but-untreated hepatitis-C-positive patients into medical care and initiate a therapy.

**Material/methods** A retrospective search within the practice management system of a single center in Germany identified among 1965 hepatitis-C-positive patients 100 untreated patients with available contact details and meeting all inclusion criteria. Patients were contacted by 2 contact rounds.

**Results** Out of 100 patients, 64% were male. Most patients (81%) were aged between 30 and 59 years. The patients belonged to high-risk groups for hepatitis C virus infections or had other comorbidities. The majority of patients injected drugs (21%) and/or were currently or had been on substitution therapy (44%); alcohol addiction was also frequent (21%). Out of 25 patients who agreed to an appointment, 10 patients (40%) started therapy and 5 additional patients (20%) agreed to therapy but were not yet able to start or had not yet made a decision. One‑third of patients who agreed to an appointment did not show up.

**Conclusions** Diagnosed-but-untreated patients are an important subgroup of hepatitis-C-positive patients; their recall to the clinic for direct-acting antiviral therapy is possible. However, inaccurate contact information, unresponsiveness to outreach, and further reluctance to attend doctor appointments limited the overall impact of this program. Regular review of the patients’ contact details may facilitate both follow-up and recall.

## Zusammenfassung

**Zielsetzung** Ziel des ReLink‑Projekts ist die Reintegration diagnostizierter aber unbehandelter Hepatitis C‑positiver Patient*innen in die medizinische Versorgung und ihre Therapie.

**Material/Methoden** Durch eine retrospektive Suche im Praxisverwaltungssystem eines einzelnen Zentrums in Deutschland wurden unter 1965 Hepatitis C-positiven Patient*innen 100 unbehandelte Patient*innen mit verfügbaren Kontaktdaten identifiziert, die alle Einschlusskriterien erfüllten. Diese wurden in zwei Runden kontaktiert.

**Ergebnisse** Von 100 Patient*innen waren 64% männlich. Das Alter der meisten Patient*innen (81%) lag zwischen 30 und 59 Jahren. Die Patient*innen gehörten Hochrisikogruppen für Hepatitis C‑Infektionen an oder hatten andere Komorbiditäten. Die Mehrheit der Patient*innen injizierte Drogen (21%) und/oder substituierte aktuell oder in der Vergangenheit (44%); Alkoholabhängigkeit war ebenfalls häufig (21% der Patient*innen). Von 25 Patient*innen, die einem Termin zustimmten, begannen 10 Patient*innen (40%) eine Therapie und fünf weitere Patient*innen (20%) stimmten einer Therapie zu, konnten sie aber noch nicht beginnen, oder waren noch unentschieden. Ein Drittel der Patient*innen mit zugesagtem Termin erschien nicht.

**Zusammenfassung** Diagnostizierte aber unbehandelte Patient*innen stellen eine wichtige Subgruppe Hepatitis C‑positiver Patient*innen dar; ihre Rückführung zur direkt wirkenden antiviralen Therapie in die Klinik ist möglich. Allerdings limitierten fehlerhafte Kontaktinformationen, Unempfänglichkeit für Kontaktaufnahmen und Zurückhaltung beim Wahrnehmen von Arztterminen die Gesamtwirkung dieses Programms. Eine regelmäßige Überprüfung der Patient*innenkontakte könnte sowohl die Nachbeobachtung als auch die Rückführung ermöglichen.

## Introduction

A hepatitis C virus (HCV) infection can be categorized as either acute (time from infection <6 months) or chronic (persisting >6 months). Chronic HCV infections (50–70% of cases) 1 may result in a detectable liver damage of varying extent 2 and extrahepatic manifestations 2 3 . In the long-term,10–30% of patients may develop liver cirrhosis leading to end-stage liver disease 1 4 or hepatocellular carcinoma (1–5% of patients with liver cirrhosis per year) 4 .

Based on recent World Health Organization (WHO) estimations, about 58 million people had a chronic hepatitis C infection worldwide in 2019 5 6 . However, the majority (79% of cases) remains unaware of their infection, and 62% of those diagnosed are treated 6 . Former obstacles, such as low coverage of screening and diagnostic services along with high treatment costs, especially in low-income countries 7 , may have been alleviated 6 . However, there is still a need to increase treatment coverage (currently 13%) to reduce the death rate due to hepatitis C 6 8 .

In Germany, approved treatment options for hepatitis C are combination therapies based on direct-acting antiviral agents (DAA), ribavirin, and historically, PEG-interferon-α 9 . Until the onset of the DAA era 10 , interferon-based therapies, prone to moderate‑to‑severe side effects 11 12 , were recommended in Germany 2 and applied in other regions as well 13 but no longer play a role in treatment regimens 9 14 . DAAs are the current standard of therapy 9 13 .

Based on therapeutic advances, in 2016, the WHO set a global goal to eliminate hepatitis C by 2030 15 . Mortality due to hepatitis C has decreased since 2019, possibly as a result of this global strategy 5 6 .

Germany will most likely not reach the 2030 target. However, efforts are being made: in 2021, a nationwide, one-time, free screening program for hepatitis B virus (HBV) and HCV infections was launched for people aged >35 years 8 16 to overcome screening deficiencies. Further major challenges include the lack of awareness among the general population and especially the limited access to or treatment of the most relevant at-risk patient groups, such as people who inject drugs (PWID), men who have sex with men, migrants, and prisoners 8 17 . In Germany, PWIDs account for 80% of the newly diagnosed HCV infections with a known transmission mode 18 .

The ReLink project aims to motivate diagnosed-but-untreated patients (DBUs) with confirmed hepatitis C diagnosis, to re-engage them in medical care, and to start hepatitis C therapy. Additionally, starting points to improve the diagnosis and treatment of HCV infection should be identified to minimize the rate of DBUs.

## Materials and Methods

The project was based on a retrospective search within a practice management system in a single center in Germany. Data analysis included the following aspects: a) number of patients per defined category in the patient flow in the evaluation period, b) demographic and social characteristics depending on availability (at least sex and age), c) the number of attempts to contact patients by e-mail or telephone, etc., and the respective result (contact, no contact, appointment arranged; see contact process for further details), and d) the number of patients who agreed to therapy.

A patient cohort of untreated hepatitis-C‑positive patients was defined based on existing patient data in the practice management system. The patient data were anonymized; therefore, no conclusion could be drawn on the individual patient. Using the search function of the practice management software, an open search for patients with confirmed hepatitis C diagnosis was conducted for visits at the center during the period from January 2019 to June 2022. This time frame was chosen with regard to data protection considerations and the treatment contract, assuming that only patients who did not visit the center for a maximum of 3 years were eligible for contact. The resulting cohort included 1965 patients. It was narrowed down to untreated patients, i.e., with no prior hepatitis C therapy, and by application of the search parameter ‘NOT’ in combination with the common hepatitis C medications ‘epclusa’, ‘harvoni’, ‘maviret’, ‘pegasys’, ‘pegintron’, ‘ribavirin’, ‘sovaldi’, ‘viekirax’, ‘vosevi’, or ‘zepatier’, resulting in a cohort of 496 patients. Through a more detailed manual search, we aimed to exclude patients who had already been treated for hepatitis C, which may not have been detected in the automated search due to writing errors in the patient file, external treatment, participation in studies, etc. This narrowed the cohort down to 196 patients, who could potentially be contacted. Additional analysis on predefined factors disqualifying patients from therapy (see exclusion criteria below) was conducted, resulting in the final patient set (FPS) of 100 patients, i.e., patients who could be contacted.

The FPS was characterized using the following patient characteristics considered for analysis: the patient *’* s sex and age (age ranges 0 to 29 years, 30 to 59 years, and ≥60 years) and with or without a migration background. In addition, factors were collected that could considerably affect the patient *’* s decision to start therapy. These factors included the comorbidity of human immunodeficiency virus infection, concomitant alcohol addiction, prior intravenous substance use, past or current substitution therapy, patient liver cirrhosis, and patient death.

### Exclusion criteria

Patients were not considered for the contact process for future therapy if a) there were aged >80 years or b) HCV-positive with further severe comorbidities that suggest a short survival time, such as metastatic carcinoma. Patients were also excluded if c) they had refused HCV therapy multiple times or d) there was evidence for poor compliance (suspicion of “insufficient adherence to therapy”) or e) patients had moved, changed doctors or were deceased or f) they were in prison and could not be reached for a visit at the center.

### Contact process

All patients included in the FPS were contacted by telephone by a specially trained and qualified person. Potential results of a telephone call had been predefined, using a data model, by which the following outcomes of the contact rounds were collected. A mixture of landline (20%) and mobile numbers (80%) was used. The second saved number was only used to contact patients who could not be reached on the first number. The categories in case the patient was reached by phone were as follows: a) appointment at the center was made, b) patient already treated, c) patient moved/ changed doctor, and d) appointment at the center impossible due to the patient’s poor physical condition. The categories in case the patients could not be reached by phone were as follows: a) no-one answered the telephone (free-line signal or mailbox), b) telephone number apparently invalid, and c) no valid telephone number exists from the outset; a further category was ‘other’.

After the first contact round, all patients who could initially not be reached were contacted a second time: Again, telephone contact was attempted first, focusing on patients who had been previously unavailable. Patients who remained unavailable by telephone were contacted in writing—depending on available data, either by an e‑mail and/or a personalized letter, with a request for callback and an appointment. Each patient was contacted through at least two different methods.

### Ethics statement

An ethics vote was not required due to the retrospective nature of this project. As only anonymized patient data were used, an explicit patient consent was not needed. The project was conducted in compliance with the current version of the Declaration of Helsinki.

## Results

**Fig. 1:**
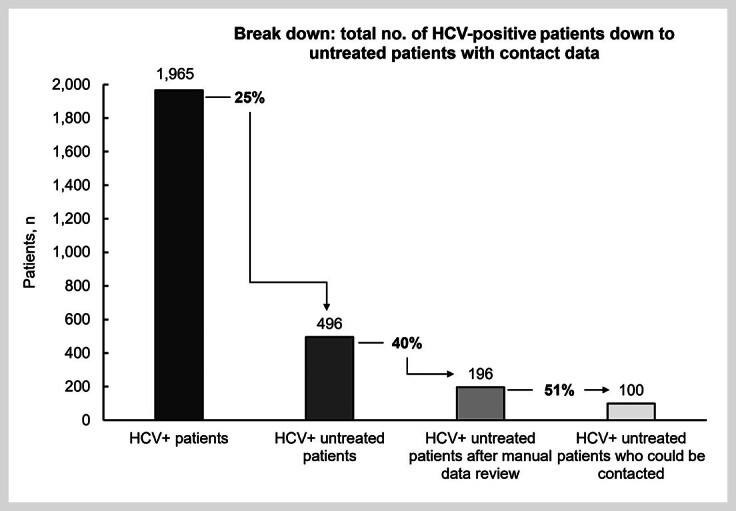
Cascade of the ReLink program: There were 1,965 hepatitis C-positive patients who visited the center between Jan 01, 2019 and Jun 30, 2022, of whom 496 patients were untreated for hepatitis C. After manual review of the patient records in the practice management system for writing errors in the patient data files, external treatment or participation in studies, 196 patients were identified; contact details were available for 100/196 patients. %: percentage share of the total number of the previous cohort; HCV+: hepatitis C-positive; n/no.: number.

The patient flow is shown in Fig. 1 depicting the size of the sample. In total, there were 1965 hepatitis-C-positive patients who visited the center between January 2019 to June 2022 and 496 patients who were diagnosed with but untreated for hepatitis C; their patient records were reviewed manually, leaving 196 patients for potential contacting. The three main reasons for exclusion of patients from the cohort of 496 patients were participation in a clinical trial, change to another doctor’s practice, and ambiguous documentation. Further analysis identified 96 patients who met predefined therapy-exclusion factors, resulting in the FPS of 100 patients with contact details. Reasons for patient exclusion were further severe comorbidities (28 patients), elderly patients (>80 years, 19 patients), multiple refusals of HCV therapy (16 patients), death (13 patients), relocation or change of doctor (11 patients), evidence for poor compliance/ difficult situations in life (5 patients), and current imprisonment (4 patients).

## Patient characteristics

Two‑thirds of patients who could be contacted were male (64%; 64/100), and the majority of patients (81%; 81/100) were aged 30 to 59 years ( Table 1 ). Nearly one-quarter (23%; 23/100) of patients had a migration background. Patients could provide several answers on comorbidities; the three most frequent responses were ‘substitution therapy’ (44%; 44/100; either current or past), intravenous substance abuse, and alcohol addiction (each 21%, 21/100 Table 1 ).

**Table:** **Table 1** Patient characteristics (FPS).

Patient characteristics	FPS (N=100) n (%)
**Age range (years)**	
0–29	1 (1)
30–59	81 (81)
≥60	18 (18)
**Sex**	
Female	36 (36)
Male	64 (64)
**Migration background**	
Yes	23 (23)
No	77 (77)
** Comorbidities ^#^**	
Substitution therapy (current or past)	44 (44)
Intravenous substance abuse	21 (21)
Alcohol addiction	21 (21)
Coinfection with HIV	14 (14)
Liver cirrhosis	13 (13)
^#^ Multiple answers were possible. FPS, final patient set; HIV, human immunodeficiency virus	

### Telephone contact

**Fig. 2:**
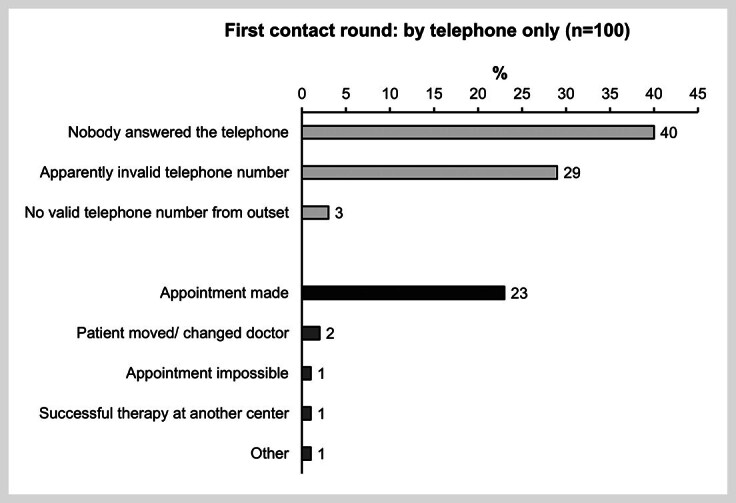
Results of the first contact round by telephone only. #Free-line signal or mailbox; §The patient’s poor physical condition prevented any appointment at the center; &The patient’s partner informed the center that the patient had died. n: number.

At first, an attempt was made to contact the patients by telephone ( Fig. 2 ), i.e., by a mixture of landline (20%) or mobile numbers (80%). The second saved number was only used for contact attempts in cases where patients who could not be reached on the first number. In 40% of the cases (40/100), there was no answer; for 29% of patients (29/100), apparently invalid phone numbers were recorded, and in 23% of the cases (23/100), the patients answered the telephone, and an appointment was scheduled.

**Fig. 3:**
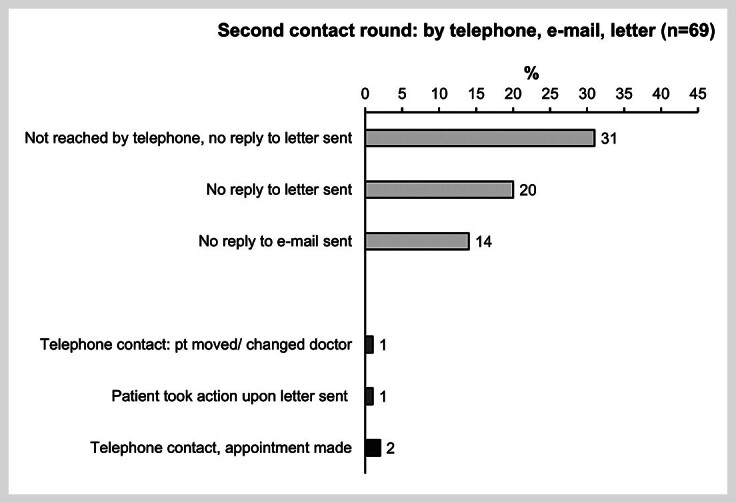
Results of the second contact round by telephone, e-mail and letter. #After receiving the letter the patient called by telephone and asked not to be contacted again. n: number; pt: patient.

In the second contacting round, we focused on patients who could not be reached previously. Telephone calls resulted in appointments for two patients (2.9%; 2/69; Fig. 3 ) only. The other patients were contacted by e-mail or letter, in most cases without response (94.2%; 65/69). Two patients who could be reached by the chosen type of contact had moved or asked not to be contacted again ( Fig. 3 ).

### Start of therapy

**Fig. 4:**
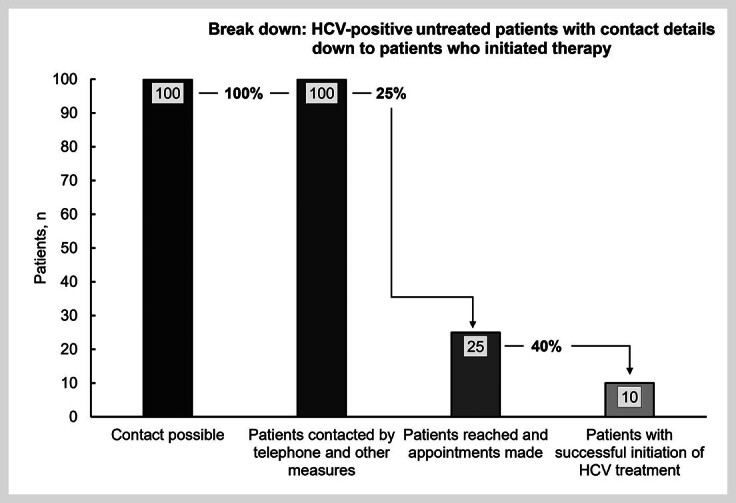
Cascade of the ReLink program of DBUs with hepatitis C (n=100) who visited the center between Jan 01, 2019 and Jun 30, 2022. All of them were contacted by several measures, i.e., telephone calls, e-mail and letters. Twenty-five of 100 patients were reached and appointments at the center made. Ten patients agreed to therapy and were treated. %: percentage share of the total number of the previous cohort; DBU: diagnosed-but-untreated patients; HCV: hepatitis C; n/no.: number.

**Fig. 5:**
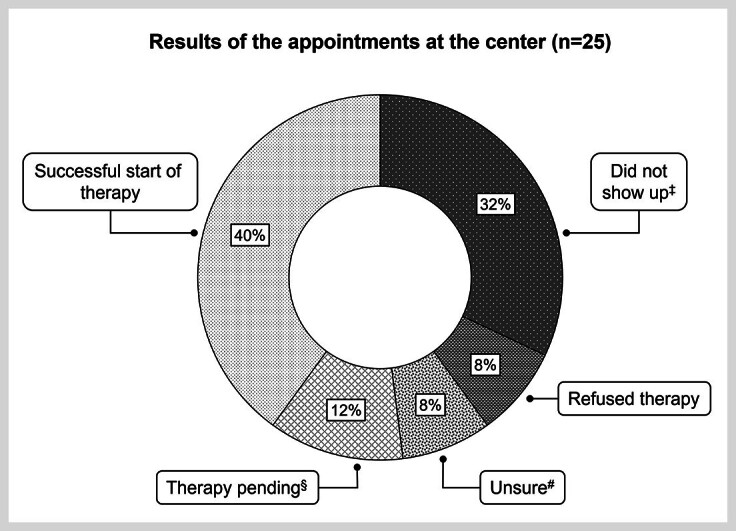
Results of appointments and agreement rate to initiation of hepatitis C therapy. ‡Did not show up for the appointment, despite having agreed to it; #Patients were undecided after the doctor’s consultation and needed time for considering a follow-up appointment; §Patients generally agreed to therapy, but the start had to be rescheduled due to other medical interventions or special situations such as pregnancy or breast feeding. n: number.

In the FPS, one-quarter of patients (25%; 25/100) had agreed to an appointment at the center ( Fig. 4 , Fig. 5 ). Ten patients (40%; 10/25) out of these successfully initiated treatment against hepatitis C infection ( Fig. 5 ). Five additional patients (20%; 5/25) could be encouraged to visit the center but have not yet initiated therapy. Among them, three patients (12%; 3/25) agreed to start anti-HCV treatment but postponed it due to pregnancy, breast feeding, or dental surgery. Initiation of therapy is planned after the medical interventions or conditions. Two patients (8%; 2/25) were still uncertain and needed time to reconsider therapy options; they will be offered therapy upon their next appointment at the center again. Two other patients categorically refused anti-HCV therapy (8%; 2/25), and nearly one‑third of patients (32%; 8/25) did not show up, despite initially agreeing to an appointment by telephone.

## Discussion

This retrospective single‑center ReLink study aimed to recall patients back to medical care and, thus, to hepatitis C therapy with the final goal of an HCV micro-elimination. While the goal of the study was similar to other ReLink studies, the methodology differed from previous studies. First, in this study, the profile of the DBUs was based on diagnostic codes, whereas in most studies, the identification criteria were based on ribonucleic acid or antibody tests. Second, the contact method in this case consisted of two phone calls, whereas in other examples of similar studies, the chosen methods were three phone calls followed by a text message 19 , two phone calls followed by a letter to the general practitioner 20 , or even five phone calls 21 . More contact activities could increase patient feedback, which should be taken into account when designing similar studies.

The number of patients who could be relinked here (25%) falls within the range observed in other ReLink projects, with 31% 22 and a randomized clinical trial (25%) 23 , or is lower than reported for other projects (50% to 74%) 21 24 . The percentage of patients who finally received treatment (40%) exceeds the reported overall DAA treatment rate (19%) for six ReLink projects 22 and is similar to a ReLink project in Latin America, being one of the six 25 , and a Spanish ReLink project (each 25%) 21 , and lower than compared to the French RECONVOCC study (40%) 24 . In this study, 8% of the patients contacted refused treatment and 32% did not show up at the center. These findings are in the range of other reports with 1% to 18% of patients refusing treatment 21 24 26 and 5% of patients lost to follow-up 24 , or 12% of patients not attending appointments 20 ; the strategies to contact patients differed between these projects.

The patient characteristics were similar to other studies, with 64% male patients in this study versus 59% 24 , 57.4% 25 , 49% 27 , or 40% 28 in other studies. The prevalence of the so-called difficult‑to‑treat risk groups, especially people with intravenous substance abuse (21% of patients), prison inmates (4% of patients), and patients with alcohol addiction (21% of patients), as well as 44% in substitution, was high in our cohort. The rate of patients who did not visit the center despite their appointment was relatively high at 32%. This may have been due to the difficult‑to‑treat risk groups 8 29 and potentially a migration background 18 29 .

Recently, the German Robert Koch-Institute reported an increase in HBV infections (by 80%) and HCV infections (by 30%) from 2019 to 2022. Potential reasons may be increased migration due to the war in Ukraine (and resulting initial HBV/HCV diagnosis), change to an electronic laboratory reporting system (and resulting duplications), and the introduction of the nationwide HBV/HCV screening program 16 30 . Still, this indicates the need to relink patients diagnosed with HCV (and HBV) back into medical care. A Spanish ReLink project addressed estimated cost reductions projected over the patient’s lifetime for the public health system, avoiding liver complications and mortality 22 . Between 2015 and 2019, initiated hepatitis C treatments increased roughly by 10-fold compared to the strategy start, with 9.4 million people diagnosed with HCV infection receiving DAA drugs 5 6 .

With this study, we were able to motivate 10 patients, equaling 40% of patients who agreed to an appointment and 10% of patients for whom contact details were available, to reintegrate back into medical care and successfully initiate treatment for hepatitis C infection. This was lower or in line with other studies 22 25 26 and adds to the WHO’s 2030 aim to nearly eliminate hepatitis C 15 . Without engagement activities, these high-risk patients may have stayed hesitant to access the normal treatment pathway. Therefore, the ReLink project results indicate the benefit of regular patient recalls: these may help to prevent several of the challenges regarding invalid or missing contact data, e.g., due to relocation. These obstacles may be less likely if patients would be contacted sooner. Regular reviews of the data practice management system may markedly improve the quality of the stored patient contact details, as unreachable patients are a major barrier to re-engaging patients in medical care 22 .

### Limitations

As part of the ReLink project, hepatitis-C-positive untreated patients were contacted who had not visited the center for a long period of time. In order to comply with treatment contracts and based on data protection considerations, only patients who visited the center between January 2019 to June 2022, were included in the selection process, thereby restricting the number of patients. Therefore, the results of the cohort presented were based on a small proportion of the total patient number at the center. The documentation of the contact options in the practice management system was insufficient, partly incomplete, or inaccurate, which made subsequent patient contacts difficult. Sending text messages, which may have increased patient feedback, was not possible for administrative/technical reasons. Unresponsiveness to outreach presented a further limitation. Regular reviews of the data stored in the system and updates by regular patient recalls would be an option to facilitate this process in the future.

## Conclusion

DBU patients are an important subgroup who can be recalled to the clinic for DAA therapy. The patient cohort of “difficult‑to‑treat risk groups” may be reluctant or difficult to motivate to make doctor appointments, due to comorbidities and further additional burdens such as alcohol addiction or substance abuse. We have successfully re-engaged 40% of DBUs in medical care, which can be rated as a success for the individual patient. Although the effort to relink these “difficult‑to‑treat” patients back to medical care may be quite large and at least twice as time‑consuming as “other” cases, it is important and necessary for the well-being of patients and also to achieve the WHO’s goal to nearly eliminate hepatitis C by 2030.
